# Mucosal advancement flap versus ligation of the inter-sphincteric fistula tract for management of trans-sphincteric perianal fistulas in the elderly: a retrospective study

**DOI:** 10.1007/s00384-025-04846-5

**Published:** 2025-03-12

**Authors:** Tamer A. A. M. Habeeb, Massimo Chiaretti, Igor A. Kryvoruchko, Antonio Pesce, Aristotelis Kechagias, Abd Al-Kareem Elias, Abdelmonem A. M. Adam, Mohamed A. Gadallah, Saad Mohamed Ali Ahmed, Ahmed Khyrallh, Mohammed H. Alsayed, Esmail Tharwat Kamel Awad, Mohammed Hassan Elshafey, Mohamed Ibrahim Abo Alsaad, Abouelatta Kh. Ali, Hamdi Elbelkasi, Mahmoud Ali Abou Zaid, Hoda A. A. Youssef, Mona Mohammad Farid Al-Zamek, Alaa Fiad, Tamer Mohamed Elshahidy, Mahmoud R. Elballat, Ahmed Kamal El Taher, Mohamed Mahmoud Mokhtar Mohamed, Ahmed Khaled AboZeid, Mohamed Ibrahim Mansour, Mahmoud Abdou Yassin, Ahmed Salah Arafa, Mohamed Lotfy, Bassam Mousa, Baher Atef, Sameh Mohamed Naguib, Ibrahim A. Heggy, Mohamed Elnemr, Mohamed Abdallah Zaitoun, Ehab Shehata AbdAllah, Mohamad S. Moussa, Abd Elwahab M. Hamed, Rasha S. Elsayed

**Affiliations:** 1 Department of General Surgery, Faculty of Medicine, Zagazig University, Zagazig, Egypt; 2 Department of General Surgery Specialties and Organ Transplant, Faculty of Pharmacy and Medicine, Sapienza Rome University, Rome, Italy; 3 Department of Surgery No. 2, Kharkiv National Medical University, Kharkiv, Ukraine; 4 Azienda Unità Sanitaria Locale Ferrara, Ferrara, Italy; 5 Department of Surgery, Athens Metropolitan General Hospital, and University of Nicosia Medical School by HEAL Academy, Athens, Greece; 6 Department of General Surgery, Faculty of Medicine, Al-Azhar University, Assuit Branch, Assuit, Egypt; 7 General Surgery Department, Faculty of Medicine, Al-Azhar University, Cairo, Egypt; 8 General Surgery Department, Faculty of Medicine, Merit University, Sohag, Egypt; 9 Misr University for Science and Technology, Cairo, Egypt; 10 Mataryia Teaching Hospital (GOTHI), Cairo, Egypt; 11 General Surgery Department, El Mahala Hepatic Insistute, Al Gharbia, El Mahala, Tanta, Egypt; 12 Department of General Surgery, Faculty of Medicine for Girls, Al Azhar University, Cairo, Egypt

**Keywords:** Trans-sphincteric perianal fistula, Mucosal advancement flap, Ligation inter-sphincteric fistula surgery, Elderly, Recurrence, Incontinence, Observational study

## Abstract

**Purpose:**

There is no consensus on the standard approach for trans-sphincteric perianal fistulas (TPAF) in the elderly population. The most commonly used sphincter-saving procedures are ligation of the inter-sphincteric fistula tract (LIFT) and mucosal advancement flap (MAF). We aimed to evaluate the incidence and risk factors for recurrence and incontinence in elderly patients with TPAF using both approaches.

**Methods:**

This retrospective study included 257 patients who underwent LIFT (136 patients) or MAF (121 patients) for de novo and cryptoglandular TPAF between July 2018 and July 2021. Recurrent fistulas were clinically and radiologically detected using MRI. Postoperative incontinence was evaluated using the Wexner score and anorectal manometry. Logistic regression analysis was used to detect the risks of recurrence and incontinence.

**Results:**

The median ages of the patients were 68 (64, 74) and 68 (65, 74) years in the LIFT and MAF groups, respectively. Higher recurrence rates were observed after LIFT (17 (12.5%)) than after MAF (13 (10.7%)), but the difference was not statistically significant (*P* = 0.662). Postoperative incontinence was observed in 18 patients (13.2%) and seven patients (5.8%) in the LIFT and MAF groups, respectively (*P* = 0.044). The predictors for fistula recurrence were smoking (OR, 75.52; 95% CI, 1.02 to 5611.35; *P* = 0.049), length of tract (OR, 17.3; 95% CI, 1.49 to 201.13; *P* = 0.023), and CD classification (OR, 7.08; 95% CI, 1.51 to 33.14; *P* = 0.013). A low Charlson comorbidity index score (≤ 5) (OR, 0.68; 95% CI, 0.47 to 0.99; *P* = 0.046) and high postoperative mean squeeze anal pressure (OR, 0.97; 95% CI, 0.95 to 0.99; *P* = 0.001) were significant factors associated with reduced risk of incontinence. In particular, LIFT was associated with a significantly higher risk of incontinence than MAF (OR, 2.089; 95% CI, 1.006 to 4.33; *P* = 0.04).

**Conclusions:**

The healing rates of MAF and LIFT procedures did not differ significantly; however, continence was significantly better after MAF. MAF should be added to the guidelines as a good option for the treatment of TPAF in elderly patients.

**Trial registration:**

The study was registered as a clinical trial www.clinicaltrials.gov (NCT06616662).

**Supplementary Information:**

The online version contains supplementary material available at 10.1007/s00384-025-04846-5.

## Introduction

A perianal fistula (PAF) is an abnormal connection between the anal canal or rectum and the perianal skin [1]. It primarily affects young males, affecting 0.86–2 cases per 10,000 individuals. PAF is higher in smokers, diabetics, and those with high body mass index (BMI). It develops after an anorectal abscess with symptoms such as itching, discharge, and pain. It is associated with morbidity, increased cost burden, and precancerous and negatively impacts the quality of life [2–4]. According to Parks’s classification, PAF may be inter-sphincteric, trans-sphincteric, supra-sphincteric, or extra-sphincteric [5]. Others classify PAF into low fistulas (subcutaneous, inter-sphincteric, or low trans-sphincteric) and high fistulas (higher trans-sphincteric, supra-sphincteric, or extra-sphincteric) [6]. The American Gastroenterological Association (AGA) defines a trans-sphincteric fistula (TPAF) as complex fistula [7].

Treatment of TPAF presents a clinical challenge. Current treatments for TPAF are based on the correct identification of the fistula tracts and internal opening, complete destruction of the tracts, preservation of anal sphincter function, and adequate fistula tract drainage. However, these approaches cannot establish effectiveness and remain undetermined because of recurrence risk and incontinence [8–16]. Sphincter-sparing options for TPAF include plug/biological mesh [8], platelet-rich plasma (PRP) alone or with other treatments [11], video-assisted anal fistula treatment (VAAFT) [10, 17, 18], FiLaC™ [13], ligation of the inter-sphincteric fistula tract (LIFT), and mucosal advancement flap (MAF) [19]. MAF has a variable healing rate, from 37 to 90% [20–26]. LIFT is another widely recognized sphincter-saving technique first described by Rojanasakul et al. Since then, LIFT has been used as a sphincter-sparing technique to repair anal fistulas [27]. The success rate of LIFT ranges from 37 to 95% [18, 28–33]. Previous studies have assessed which treatment, MAF or LIFT, is best for high PAF and have shown no significant differences in overall success [34]. Regarding postoperative incontinence, minor incontinence in 6% of patients following LIFT was reported [35], whereas MAF repair showed no incontinence in one study [20] and incontinence ranging between 13.3 and 18.9% in other studies [24, 26, 36].

The risk factors for recurrent PAF are complex and diverse, including the anatomy of PAF, comorbidities, lack of preoperative assessment, surgeon experience, inadequate operative choice, inefficient postoperative care [37], body mass index > 25.0 kg/m^2^, high salt intake, diabetes mellitus, anorectal surgery, hyperlipidemia, spicy food, smoking, alcohol consumption [38], young age, and female sex [4]. Risk factors for postoperative incontinence include high PAF, type of surgery, and previous fistula surgery [39, 40].

Surgical management of TPAF in the elderly is challenging, and the ideal approach remains uncertain, even among experienced colorectal surgeons. Our study evaluated the incidence and risk factors of recurrence after LIFT and MAF for the treatment of TPAF in the elderly 3 years after surgery. The secondary purpose was to assess the incidence of anal incontinence and risk variables 3 years after surgery using a validated score.

## Materials and methods

### Study design and eligibility criteria

**Fig. 1:**
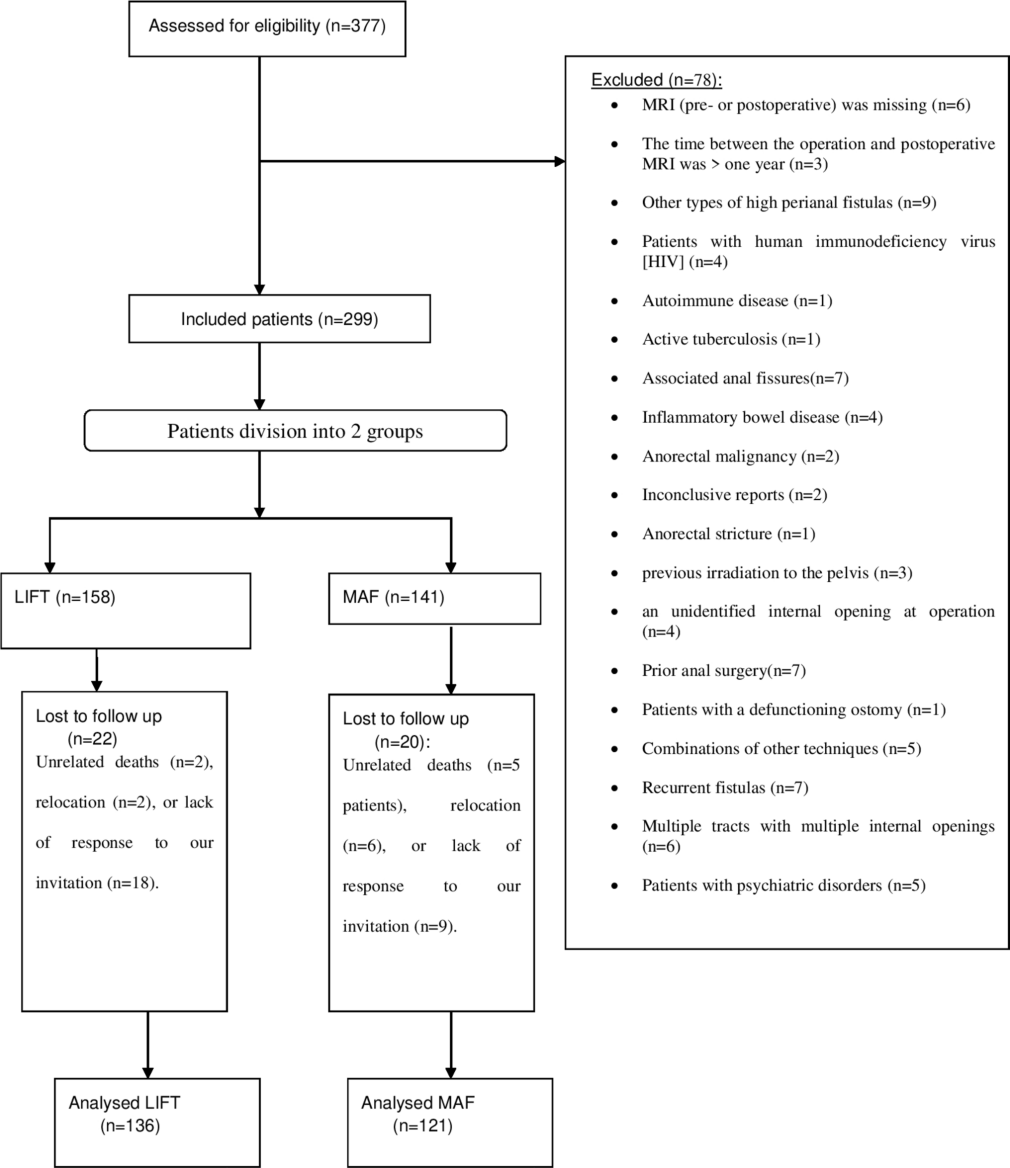
Flow chart of inclusion and exclusion criteria of studied patients

Data were retrospectively collected from 257 patients who underwent LIFT (136 patients) or MAF (121 patients) for de novo and cryptoglandular TPAF between July 2018 and July 2021. Data were retrospectively obtained using a database management system comprising surgery and physical examination notes derived from personal identities from the hospital’s electronic records. Elderly patients aged ≥ 60 years (https://www.who.int/news-room/fact-sheets/detail/ageing-and-health) who underwent LIFT and MAF as the initial definitive repair of their TPAF with or without setons were included. Figure 1 shows a flow chart of the study patients’ inclusion and exclusion criteria. This study follows the STROCSS guidelines (Strengthening the Reporting of Cohort, Cross-sectional, and Case–Control Studies in Surgery) [41].

### Participating centers

The study was conducted in four centers in Egypt and involved two academic centers (Zagazig University and Al-Azhar University) and two non-academic centers (Mataryia Teaching Hospital and El Mahala Institute).

### Outcome definitions and measurements

The study’s primary endpoint was fistula recurrence and its risk factors; the secondary endpoint was postoperative incontinence and its predictors. TPAF was classified based on endoanal ultrasound findings as low, mid, or high [42]. All patients underwent evaluation, including medical history, clinical examination, and proctoscopy to exclude specific pathologies. Clinical evaluation was confirmed preoperatively using pelvic magnetic resonance imaging (MRI) [43], high-resolution and high-definition anorectal manometry (Hawk type 2050; B-K Medical, Naerum, Denmark) [44], and three-dimensional endoanal ultrasonography (3D-EAUS) [45]. A knotted loose seton was often placed prior to definitive treatment to promote drainage and reduce anorectal infection load in the workup prior to the definitive procedure. Primary healing was determined clinically by the closure of the external opening, absence of clinical symptoms, and radiologically defined as a fibrotic tract on MRI [43]. An unhealed fistula is characterized by recurrence of the external opening following initial healing, reappearance of prior symptoms (such as drainage and inflammation), or continued presence of the fistula (termed a persistent fistula) after 12 weeks [32]. The time to recurrence was defined as the time from healing to when the patient’s file documented recurring PAF symptoms, reopening of the external opening, or new primary fistula formation (including transformation to an inter-sphincteric fistula tract after LIFT). MRI was performed to confirm the presence of recurrent PAF. Incontinence was determined using the Wexner score (Cleveland Clinic Florida Fecal Incontinence Severity Scoring System) for all patients preoperatively and 6 months postoperatively [46]. Postoperative complications were graded according to the Clavien-Dindo (CD) classification [47].

### Preoperative preparation and surgical procedures

An experienced anesthetist evaluated all patients. On the morning of surgery, all patients received a single phosphate enema. During anesthesia induction, a single dose of a third-generation cephalosporin (1 g cefotaxime) was administered. Surgery was performed during daycare combined with seton removal, if present. The procedures were performed in a lithotomy or jackknife-prone position. LIFT was performed as previously described [48]. Correct tract ligation was confirmed by injecting hydrogen peroxide through an external opening to detect extravasation. The defect in the external anal sphincter was sutured using 3–0 polyglactin. The perianal wound was not closed and was covered daily with dry gauze. The external opening was excised, and the fistulous tract was curetted to the level of the external sphincter. MAF was performed as previously described [49]. We used Park’s anal retractor or Scott’s anal retractor according to its availability [50]. Following demarcation of the fistula course, the fistula tract was curetted, and the scar tissue around the external opening was excised. The internal opening was closed using interrupted 3–0 absorbable sutures. The base of the advancement flap is sufficiently wide to ensure adequate circulation. The treatment choice was based on the choice of a specialized colorectal surgeon and the availability of logistics such as Park’s anal retractor or Scott’s anal retractor.

### Postoperative care and outcome follow-up

Postoperative care and pain control were associated with enhanced recovery after surgery [51]. All patients were discharged 1 day after surgery and were instructed to use sitz baths twice daily. The patients were administered intravenous third-generation cephalosporin for 1 day following surgery and oral antibiotics (ciprofloxacin 500 mg and metronidazole 500 mg) for up to 1 week, depending only on the severity of the inflammatory changes in the perianal area and were prescribed individually. Liquid food intake was resumed in the evening after the operation, and the patients were instructed to adhere to a soft diet for the next 2 days. Wound dressing on the second postoperative day. Postoperative follow-up involved assessing healing and recurrence through scheduled outpatient appointments at 1, 4, and 6 weeks and 3 and 6 months, continuing annually until the end of the follow-up period or upon patient-initiated contact regarding recurrence or symptom exacerbation. MRI was conducted again if recurrence occurred clinically or at the conclusion of the study when complete clinical healing was noted. Telephone interviews were conducted to assess the condition of the fistulas in patients who did not participate in the outpatient clinical follow-up. The operating surgeon or assistant surgeons conducted postoperative interviews and examinations. Endoanal ultrasonography and manometry were performed at 12 months postoperatively. After 6 months, a comprehensive evaluation of anal continence was conducted through a thorough clinical examination and application of the Wexner score.

### Statistical analysis

All statistical analyses were performed using the SPSS version 28 (IBM Corp., Armonk, NY, USA). Continuous parametric data are presented as the mean and standard deviation (SD), and the data were analyzed between the two groups using an unpaired *t*-test. Continuous non-parametric data were presented as the median and interquartile range (IQR) and were analyzed using the Mann–Whitney *U* test. Categorical data are presented as frequencies and percentages and were analyzed using the appropriate chi-squared or exact test. Logistic regression was used to assess different factors associated with the incidence of recurrence and incontinence. Only variables with a *P*-value < 0.2 in the univariate analysis were included in the multivariable analysis. We can not include all variables because of sample size restrictions. Statistical significance was defined as a two-tailed *P*-value of < 0.05.

## Results

Table 1 shows the demographic, clinical, and radiological (MRI, ultrasound) characteristics of the studied groups. There were no statistically significant differences between the two groups, except for the frailty index (*P* = 0.002), American Society of Anesthesiology (ASA) score (*P* = 0.03), and time from abscess drainage to TPAF diagnosis (*P* = 0.014). The median age of the patients at surgery was 68 (64, 74) years and 68 (65, 74) years, and 90 (66.2%) and 80 (66.1%) were male in both groups, respectively. The assessed patients completed follow-up for up to 3 years. All patients had a single internal opening which 90 (66.2%) and 75 (62%) were located at the dentate line, 26 (19.1%) and 29 (24%) above the dentate line, and 20 (14.7%) and 17 (14%) below the dentate line in both groups, respectively. High TPAF was the most common form in both groups: 99 (72.8%) and 85 (70.2%) patients.

**Table 1:** Demographic, clinical, and radiological (MRI, ultrasound) characteristics of the studied groups

Item	LIFT group (*n* = 136)	MAF group (*n* = 121)	*P*-value
Age, years (median, IQR)	68 (64, 74)	68 (65, 74)	0.339
BMI, kg/m^2^ (median, IQR)	27.5 (25, 33)	31 (26, 33)	0.110
Sex (*n*, %)			
Male	90 (66.2%)	80 (66.1%)	0.992
Female	46 (33.8%)	41 (33.9%)	
Vaginal delivery (*n*, %)	24 (52.2%)	18 (43.9%)	0.441
*N* of vaginal deliveries (median, IQR)	2 (2, 3)	2 (1.75, 3)	0.706
Traumatic injury during vaginal delivery (episiotomy or perineal tear) (*n*, %)	14 (58.3%)	12 (66.7%)	0.582
Smoking (*n*, %)	21 (15.4%)	28 (23.1%)	0.117
Hypertension (*n*, %)	26 (19.1%)	16 (13.2%)	0.202
DM (*n*, %)	24 (17.6%)	21 (17.4%)	0.951
CHD (*n*, %)	21 (15.4%)	19 (15.7%)	0.954
Frailty index (*n*, %)			
Severely frail	14 (10.3%)	23 (19%)	**0.002**
Moderately frail	18 (13.2%)	35 (28.9%)	
Mildly frail	49 (36%)	23 (19%)	
Vulnerable	29 (21.3%)	19 (15.7%)	
Managing well	18 (13.2%)	13 (10.7%)	
Well	8 (5.9%)	8 (6.6%)	
Charlson comorbidity index score (median, IQR)	6 (5, 8)	6 (5, 8)	0.764
4	6 (4.4%)	5 (4.1%)	0.720
5	42 (30.9%)	40 (33.1%)	
6	39 (28.7%)	26 (21.5%)	
7	14 (10.3%)	17 (14%)	
8	17 (12.5%)	14 (11.6%)	
9	11 (8.1%)	10 (8.3%)	
10	0 (0%)	2 (1.7%)	
11	7 (5.1%)	7 (5.8%)	
ASA physical status (*n*, %)			
I	6 (4.4%)	18 (14.9%)	**0.03**
II	40 (29.4%)	30 (24.8%)	
III	71 (52.2%)	61 (50.4%)	
IV	19 (14%)	12 (9.9%)	
Symptoms of primary fistula before surgery (*n*, %)			
Discharge and itching	56 (41.2%)	50 (41.3%)	0.233
Pain	38 (27.9%)	24 (19.8%)	
Pruritus	42 (30.9%)	47 (38.8%)	
Aetiology of primary fistula (*n*, %)			
Denovo	56 (41.2%)	44 (36.4%)	0.430
Following abscess drainage	80 (58.8%)	77 (63.6%)	
Time from abscess drainage till fistula diagnosis, months (median, IQR)	5.05 (3.9, 6)	4.1 (2.45, 5.2)	**0.014**
Time from fistula diagnosis till surgery, months (median, IQR)	4 (3, 6)	4.1 (3, 6)	0.725
Sphincter defect (*n*, %)	25 (18.4%)	20 (16.5%)	0.696
Number of tracts (*n*, %)			
Single and unbranched	106 (77.9%)	99 (81.8%)	0.440
Single and branched	30 (22.1%)	22 (18.2%)	
Position of internal opening (*n*, %)			
Below dentate line	20 (14.7%)	17 (14%)	0.638
At dentate line	90 (66.2%)	75 (62%)	
Above dentate line	26 (19.1%)	29 (24%)	
Position of external opening (*n*, %)			
Anterior	59 (43.4%)	48 (39.7%)	0.910
Posterior	39 (28.7%)	39 (32.2%)	
Right lateral	19 (14%)	18 (14.9%)	
Left lateral	19 (14%)	16 (13.2%)	
Horseshoe extension (*n*, %)	26 (19.1%)	22 (18.2%)	0.848
Distance between external opening and anal verge, cm (median, IQR)	4.1 (3.63, 4.98)	4.1 (3.7, 4.3)	0.899
Type of trans-sphincteric fistula (*n*, %)			
Low	16 (11.8%)	16 (13.2%)	0.898
Mid	21 (15.4%)	20 (16.5%)	
High	99 (72.8%)	85 (70.2%)	
Length of tract, cm (median, IQR)	4.9 (4.5, 5.5)	4.9 (4.6, 5.7)	0.695
Fistula diameter, mm (median, IQR)	1.2 (1, 2)	1.2 (1, 2)	0.405
Inflammatory changes (*n*, %)			
Absent	92 (67.6%)	81 (66.9%)	0.917
Focal collection (abscess)	19 (14%)	19 (15.7%)	
Diffuse inflammation	25 (18.4%)	21 (17.4%)	
Size of focal collection (abscess), mm (*n*, %)	25.79 (5.54)	29.58 (8.46)	0.113
Draining seton (*n*, %)	19 (14%)	19 (15.7%)	0.696
Time from seton insertion till surgery, weeks (median, IQR)	12 (8, 16)	10.5 (7.75, 14.5)	0.620

Numerical data are presented as mean (SD) or median (IQR) as appropriate, and categorical data are presented as frequency (%), statistical significance at *P*-value < 0.05

*BMI* body mass index, *DM* diabetes mellitus, *CHD* coronary heart disease, *ASA* the American Society of Anesthesiologists

Intraoperative and postoperative data are shown in Table 2. There were no statistically significant differences between the groups, except that the median operation time was longer (34 (28, 44) vs. 27 (23, 30) min (*P* < 0.001)), with higher Clavien-Dindo classification Grade III (13 (10.7%) vs 3 (2.2%), *P* < 0.001)), and lower median postoperative visual analog scale (4 (3, 5) vs. 6 (5, 7) (*P* < 0.001)) in the MAF group than in the LIFT group. No intraoperative complications were observed in either of the groups. Twelve (10%) patients who underwent MAF technique required reoperation under spinal anesthesia to treat postoperative complications without further complications after dealing with these complications.

**Table 2:** Intraoperative and postoperative data of the studied groups

Item	LIFT group (*n* = 136)	MAF group (*n* = 121)	*P*-value
Abscess detected during operation (*n*, %)	8 (5.9%)	6 (5%)	0.745
Operative time, min (median, IQR)	27 (23, 30)	34 (28, 44)	**< 0.001**
Postoperative hospitalization, days (median, IQR)	3 (2, 4)	3 (2, 4)	0.206
Postoperative complications (*n*, %)			
No complications	117 (86%)	105 (86.8%)	0.099
Bleeding	4 (2.9%)	0 (0%)	
Flap retraction	0 (0%)	2 (1.7%)	
Perianal edema	3 (2.2%)	2 (1.7%)	
Submucosal hematoma	0 (0%)	3 (2.5%)	
Urinary retention	3 (2.2%)	1 (0.8%)	
Wound infection	9 (6.6%)	8 (6.6%)	
Clavien-Dindo classification (*n*, %)			
Grade 0	117 (86%)	105 (86.8%)	**< 0.001**
Grade I	4 (2.9%)	0 (0%)	
Grade II	12 (8.8%)	3 (2.5%)	
Grade III	3 (2.2%)	13 (10.7%)	
Treatment of postoperative complications (*n*, %)			
No treatment	117 (86%)	105 (86.8%)	**< 0.001**
Anti-edema and antibiotic	3 (2.2%)	2 (1.7%)	
Drainage of infection under anesthesia and antibiotic	0 (0%)	7 (5.8%)	
Evacuation of hematoma under anesthesia and antibiotic	0 (0%)	3 (2.5%)	
Open wound in bed to drain infection and antibiotic	9 (6.6%)	1 (0.8%)	
Revision of the flap under anesthesia	0 (0%)	2 (1.7%)	
Urinary catheterization	3 (2.2%)	1 (0.8%)	
Wound compression in bed	4 (2.9%)	0 (0%)	
Postoperative visual analogue scale (median, IQR)	6 (5, 7)	4 (3, 5)	**< 0.001**

Numerical data are presented as mean (SD) or median (IQR) as appropriate, and categorical data are presented as frequency (%), statistical significance at *P*-value < 0.05

**Fig. 2:**
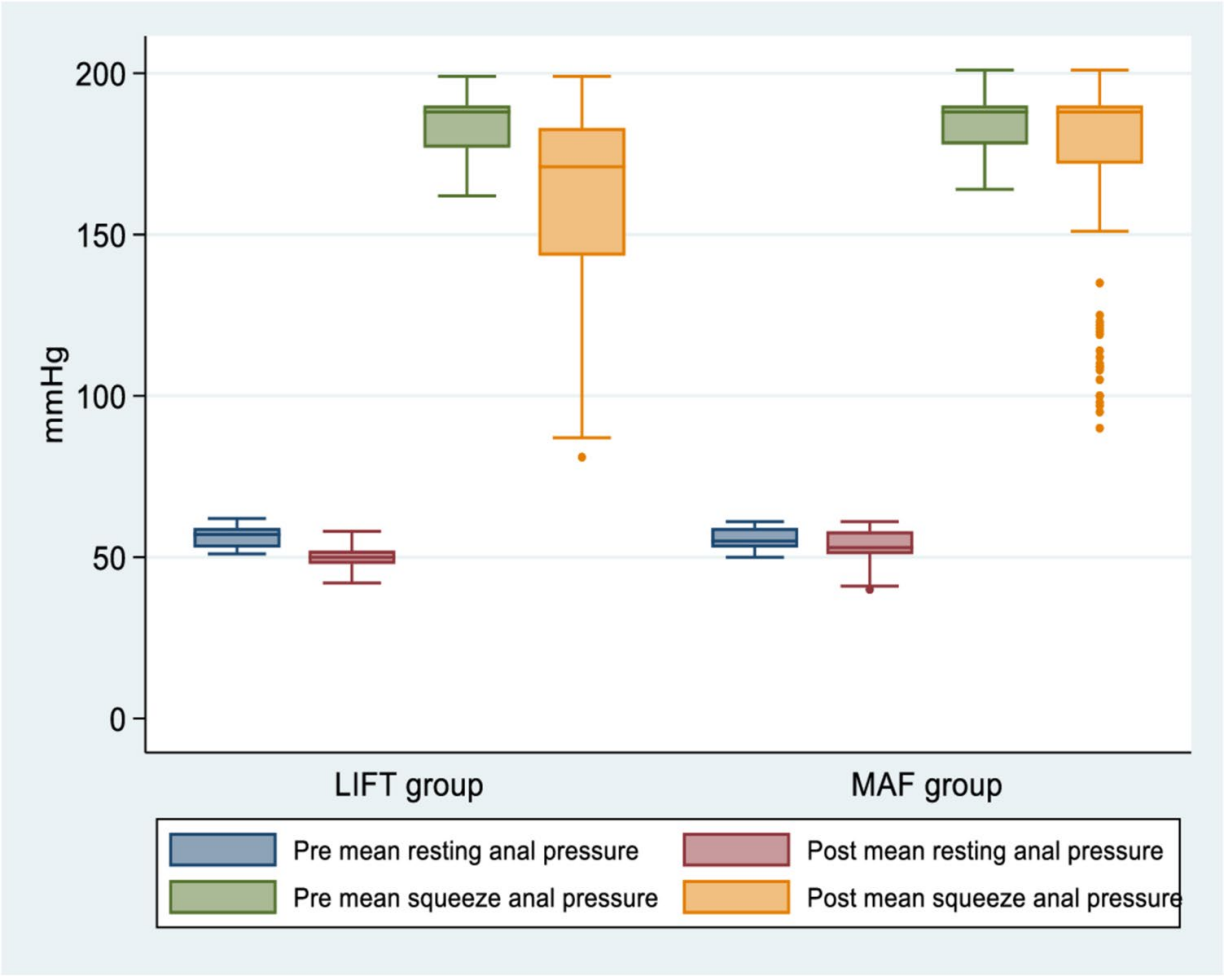
Boxplot demonstrating pre and postoperative 3D-high definition anorectal manometry results of groups

**Fig. 3:**
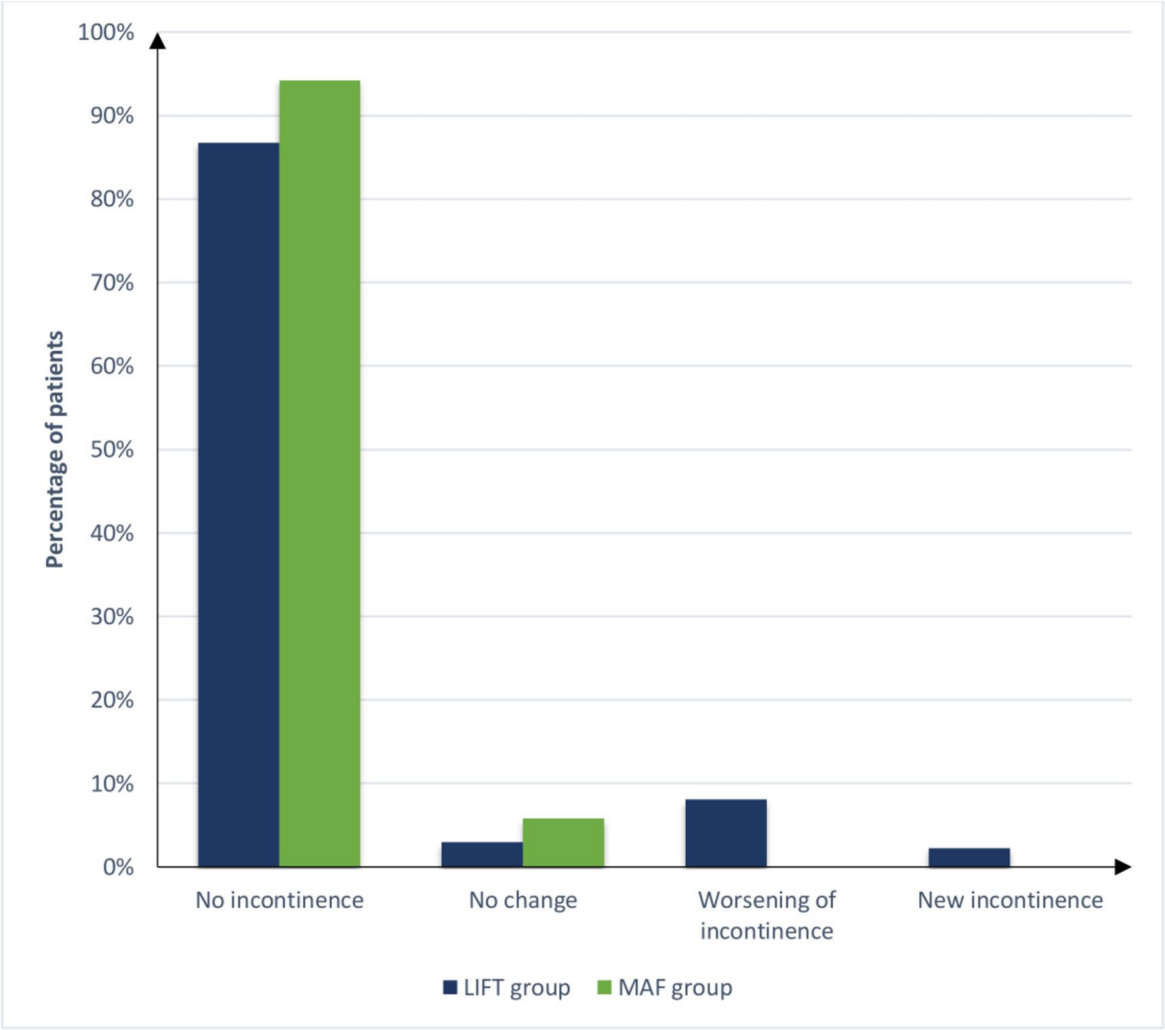
Bar chart for postoperative incontinence forms and severity in groups

**Fig. 4:**
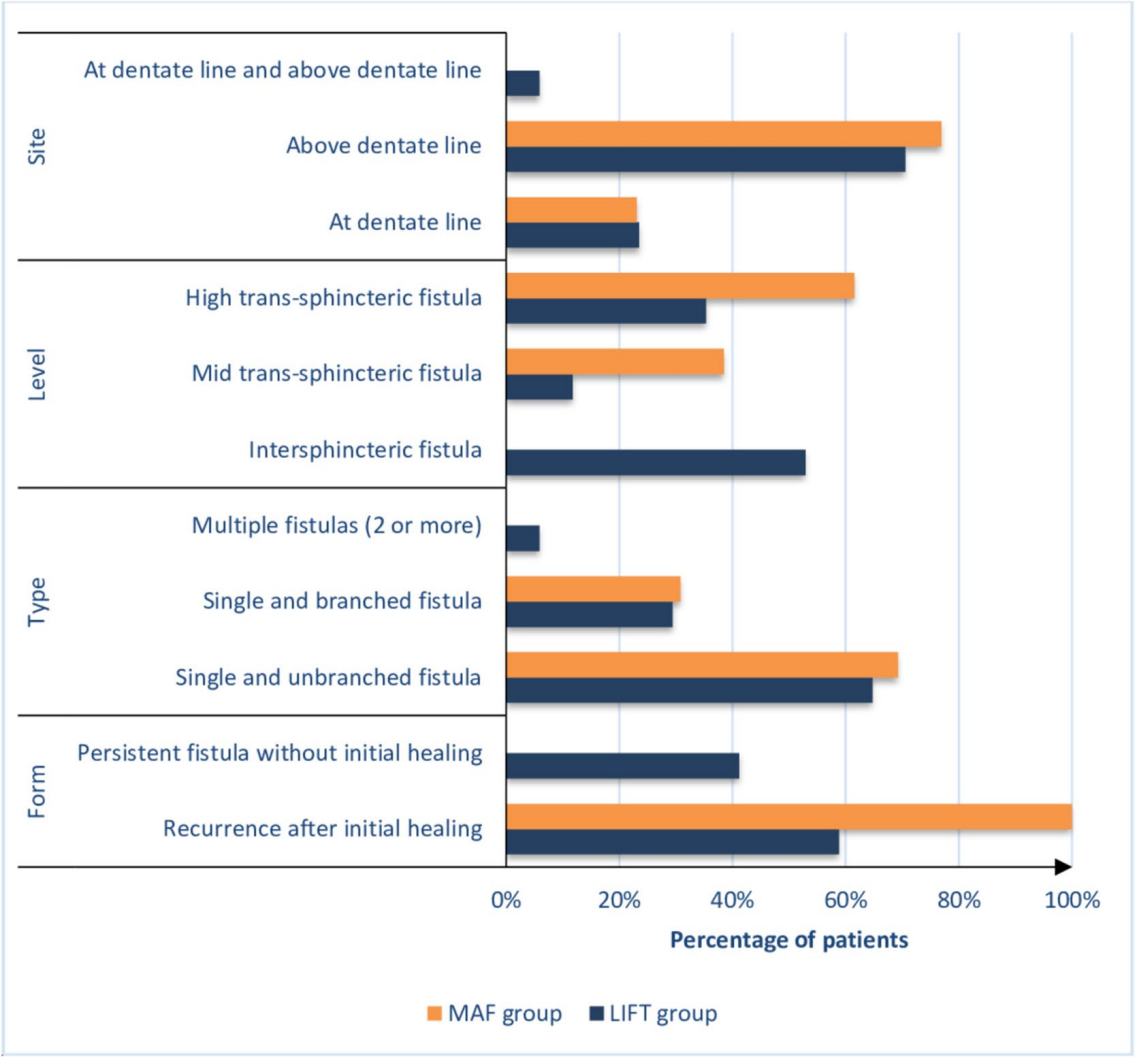
Bar chart for form, type, level, and site of recurrent fistulas in groups

Table 3 and Figs. 2, 3, and 4 show primary and secondary outcomes after surgery. At the end of the 3-year follow-up, a trend was observed towards higher recurrence rates after LIFT (17 (12.5%)) than after MAF (13 (10.7%)), but the difference was not statistically significant (*P* = 0.662). Recurrence was significantly faster after LIFT than after MAF (4.2 vs 8.9 months; *P* < 0.001). Twelve of 17 patients (70.6%) who did not heal in the LIFT group and all patients with recurrence after the MAF procedure did not prefer further treatment for recurrence. Preoperative 3D-high-definition anorectal manometry showed no statistically significant difference between the groups regarding MRP and MSP (*P* = 0.139 and *P* = 0.292, respectively). Postoperative 3D-high-definition anorectal manometry showed that the median MRP (53 (51, 58) vs. 50 (48, 52) mmHg) and median MSP (188 (172, 190) vs. 171 (142.75, 183) mmHg) were significantly higher in the MAF (*P* < 0.001; *P* < 0.001). Based on 3D-high-definition anorectal manometry, preoperative incontinence was observed in 15 patients (11%) who underwent the LIFT approach and seven patients (5.8%) who underwent the MAF approach, while postoperative incontinence was observed in 18 patients (13.2%) who underwent the LIFT approach and seven patients (5.8%) who underwent the MAF approach. Further evaluation of forms and severity of postoperative incontinence by manometric measurement showed that forms and severity of postoperative incontinence were significantly higher in the LIFT approach (*P* < 0.001). In the LIFT technique, there was no change in postoperative incontinence compared to preoperative incontinence in 4 patients (2.9%), postoperative worsening of continence compared to preoperative incontinence in 11 patients (8.1%), and the new appearance of postoperative incontinence in 3 patients (2.2%); in the MAF approach, there was no change in postoperative incontinence in any of the patients. Cleveland Clinic (Wexner) fecal incontinence scores were not significantly different pre- (*P* = 0.326) and postoperatively (*P* = 0.116), with ranges of (0–15) and (0–16) in the LIFT and MAF groups, respectively, and medians of 0.

**Table 3:** Primary and secondary outcomes of the studied groups

Item		LIFT Group	MAF Group		*P*-value	
A. Healing and recurrence						
		**(*n* = 119)**	**(*n* = 108)**			
Time from surgery till healing, weeks (median, IQR)		5.1 (4.1, 7.3)	2.1 (1.9, 4.53)		**< 0.001**	
< 6 weeks		68 (57.1%)	93 (86.1%)		**< 0.001**	
> 6 weeks		51 (42.9%)	15 (13.9%)			
		**(*n* = 136)**	**(*n* = 121)**			
Success rate of healing (*n*, %)						
Failure		17 (12.5%)	13 (10.7%)		0.662	
Success		119 (87.5%)	108 (89.3%)			
Recurrence (*n*, %)		17 (12.5%)	13 (10.7%)		0.662	
		**(*n* = 17)**	**(*n* = 13)**			
Time from surgery to diagnosis of recurrence, months (median, IQR)		4.2 (3.95, 5.05)	8.9 (7.9, 9.6)		**< 0.001**	
Forms of recurrent fistula (*n*, %)						
Recurrence after initial healing		10 (58.8%)	13 (100%)		**0.010**	
Persistent fistula without initial healing		7 (41.2%)	0 (0%)			
Length of recurrent fistula, cm (mean ± SD)		4.75 (0.71)	5.57 (0.99)		**0.014**	
Distance between external opening of recurrent fistula and anal verge, cm (mean ± SD)		2.73 (0.45)	4.32 (0.66)		**< 0.001**	
Diameter of recurrent fistula, mm (median, IQR)		0.9 (0.61, 1.25)	0.4 (0.4, 1.15)		0.086	
Type of recurrent fistula (*n*, %)						
Single and unbranched fistula		11 (64.7%)	9 (69.2%)		> 0.999	
Single and branched fistula		5 (29.4%)	4 (30.8%)			
Multiple fistulas (2 or more)		1 (5.9%)	0 (0%)			
Level of recurrent fistula (*n*, %)						
Intersphincteric fistula		9 (52.9%)	0 (0%)		**0.005**	
Mid trans-sphincteric fistula		2 (11.8%)	5 (38.5%)			
High trans-sphincteric fistula		6 (35.3%)	8 (61.5%)			
Site of internal opening of recurrent fistula (*n*, %)						
At dentate line		4 (23.5%)	3 (23.1%)		> 0.999	
Above dentate line		12 (70.6%)	10 (76.9%)			
At dentate line and above dentate line		1 (5.9%)	0 (0%)			
Number of recurrent internal opening (median, IQR)		1 (1, 1)	1 (1, 1)		0.805	
Site of recurrent external opening (*n*, %)						
Anterior		6 (35.3%)	1 (7.7%)		**0.008**	
Posterior		3 (17.6%)	6 (46.2%)			
Right lateral		0 (0%)	2 (15.4%)			
Left lateral		2 (11.8%)	0 (0%)			
Anterior and right lateral		4 (23.5%)	0 (0%)			
Anterior and left lateral		2 (11.8%)	1 (7.7%)			
Posterior and right lateral		0 (0%)	2 (15.4%)			
Posterior and left lateral		0 (0%)	1 (7.7%)			
Number of recurrent external openings (median, IQR)		1 (1, 2)	1 (1, 2)		0.837	
Treatment of recurrent fistula (*n*, %)						
Fistulotomy with no recurrence		5 (29.4%)	0 (0%)		0.052	
Refuse further treatment		12 (70.6%)	13 (100%)			
Patient satisfaction with surgery (*n*, %)		**(*n* = 136)**	**(*n* = 121)**			
Dissatisfied		17 (12.5%)	14 (11.6%)		0.697	
No response		10 (7.4%)	6 (5%)			
Satisfied		109 (80.1%)	101 (83.5%)			
B. Incontinence						
Preoperative 3D-high definition anorectal manometry (median, IQR)						
Mean resting anal pressure, mmHg	57 (53, 59)			55 (53, 59)		0.139
Mean squeeze anal pressure, mmHg	188 (177, 190)			188 (178, 190)		0.292
Postoperative 3D-high definition anorectal manometry (median, IQR)						
Mean resting anal pressure, mmHg	50 (48, 52)			53 (51, 58)		**< 0.001**
Mean squeeze anal pressure, mmHg	171 (142.75, 183)			188 (172, 190)		**< 0.001**
Preoperative incontinence (*n*, %)	15 (11%)			7 (5.8%)		0.134
Postoperative incontinence (*n*, %)	18 (13.2%)			7 (5.8%)		**0.044**
Forms and severity of postoperative incontinence (*n*, %)						
No change in postoperative incontinence compared to preoperative incontinence	4 (2.9%)			7 (5.8%)		**0.001**
Postoperative worsening of continence compared to preoperative incontinence	11 (8.1%)			0 (0%)		
New appearance of postoperative incontinence	3 (2.2%)			0 (0%)		
Preoperative Cleveland Clinic (Wexner) fecal incontinence scoring (median, IQR)	0 (0, 0)			0 (0, 0)		0.326
Postoperative Cleveland Clinic (Wexner) fecal incontinence scoring (median, IQR)	0 (0, 0)			0 (0, 0)		0.116

Numerical data are presented as mean (SD) or median (IQR) as appropriate, and categorical data are presented as frequency (%), statistical significance at *P*-value < 0.05

Logistic regression for recurrence is tabulated in Table 4. In multivariate analysis, the predictors for fistula recurrence were smoking (odds ratio (OR), 75.52; 95% confidence interval (CI), 1.02 to 5611.35; *P* = 0.049), length of tract (OR, 17.3; 95% CI, 1.49 to 201.13; *P* = 0.023), and CD classification (OR, 7.08; 95% CI, 1.51 to 33.14; *P* = 0.013). Age was not a risk factor for recurrence (OR, 1.04; 95% CI, 0.83 to 1.31; *P* = 0.724). Finally, no significant difference was observed in the risk of recurrence between the LIFT and MAF groups.

**Table 4:** Logistic regression analysis for factors associated with the incidence of recurrence

Item	Univariate analysis			Multivariable analysis		
	Unadjusted OR	95% CI	*P*-value	Adjusted OR	95% CI	*P*-value
Age, years	1.13	1.06 to 1.21	**< 0.001**	1.04	0.83 to 1.31	0.724
Sex						
Male	Ref			Ref		
Female	6.85	2.9 to 16.18	**< 0.001**	3.94	0.05 to 314.37	0.54
Smoking	16.58	6.91 to 39.8	**< 0.001**	75.52	1.02 to 5611.35	**0.049**
Charlson comorbidity index score	1.06	0.86 to 1.31	0.592			
Frailty index	1.44	0.43 to 4.82	0.55			
ASA physical status						
I	Ref					
II	2.97	0.35 to 25.05	0.318			
III	3.63	0.46 to 28.58	0.22			
IV	2.46	0.24 to 25.31	0.448			
Aetiology of primary fistula						
Denovo	Ref					
Following abscess drainage	0.7	0.32 to 1.5	0.356			
Time from abscess drainage till fistula diagnosis, months	0.93	0.76 to 1.14	0.508			
Time from fistula diagnosis till surgery, months	1.38	1.23 to 1.54	**< 0.001**	1.54	0.84 to 2.84	0.163
Draining seton	8.87	3.85 to 20.45	**< 0.001**	3.78	0.02 to 814.74	0.627
Time from seton insertion till surgery, weeks	1.05	0.91 to 1.2	0.517			
Number of tracts						
Single and unbranched	Ref			Ref		
Single and branched	12.19	5.23 to 28.4	**< 0.001**	1.2	0.01 to 241.3	0.945
Position of external opening						
Anterior	Ref					
Posterior	1.45	0.63 to 3.32	0.384			
Right lateral	0.41	0.09 to 1.92	0.26			
Left lateral	0.44	0.09 to 2.05	0.294			
Horseshoe extension	17.28	7.18 to 41.6	**< 0.001**	0.99	0.03 to 32.82	0.994
Distance between external opening and anal verge, cm	6.16	3.19 to 11.91	**< 0.001**	0.46	0.05 to 4.35	0.5
Length of tract, cm	5.64	3.04 to 10.45	**< 0.001**	17.3	1.49 to 201.13	**0.023**
Fistula diameter, mm	0.13	0.05 to 0.33	**< 0.001**	0.42	0.01 to 14.33	0.631
Operative time, min	0.98	0.94 to 1.03	0.493			
Clavien-Dindo classification	5.64	3.58 to 8.89	**< 0.001**	7.08	1.51 to 33.14	**0.013**
Postoperative VAS	1.04	0.86 to 1.26	0.655			
Mean resting anal pressure, mmHg	0.7	0.62 to 0.78	**< 0.001**	0.69	0.38 to 1.27	0.232
Mean squeeze anal pressure, mmHg	0.94	0.93 to 0.96	**< 0.001**	0.98	0.91 to 1.05	0.525
Postoperative incontinence	1.04	0.29 to 3.69	0.957			
Procedure						
LIFT	Ref					
MAF	0.84	0.39 to 1.82	0.662			

Statistical significance at *P*-value < 0.05

*OR* odds ratio, *CI* **c**onfidence interval

Logistic regression for incontinence is tabulated in Table 5. In the multivariable analysis, low Charlson comorbidity index score (≤ 5) (OR, 0.68; 95% CI, 0.47 to 0.99; *P* = 0.046) and high postoperative mean squeeze anal pressure (OR, 0.97; 95% CI, 0.95 to 0.99; *P* = 0.001) were significantly associated with risk reduction of incontinence. In particular, LIFT was associated with a significantly higher risk of incontinence than the MAF technique (OR, 2.089; 95% CI, 1.006 to 4.33; *P* = 0.04). Age was not a risk factor for incontinence (OR, 1.01; 95% CI, 0.93 to 1.1; *P* = 0.827).

**Table 5:** Logistic regression analysis for factors associated with the incidence of incontinence

Item	Univariate analysis			Multivariable analysis		
	Unadjusted OR	95% CI	*P*-value	Adjusted OR	95% CI	*P*-value
Age, years	1.06	0.99 to 1.14	0.077	1.01	0.93 to 1.1	0.827
Sex						
Male	Ref					
Female	1.61	0.7 to 3.72	0.262			
Traumatic injury during vaginal delivery (episiotomy or perineal tear)	0.91	0.14 to 6.16	0.926			
Smoking	0.79	0.26 to 2.42	0.682			
Frailty index	0.87	0.64–1.16	0.354			
Low Charlson comorbidity index score (≤ 5)	0.72	0.53 to 0.98	**0.038**	0.68	0.47 to 0.99	**0.046**
ASA physical status						
I	Ref			Ref		
II	1.77	0.2 to 15.95	0.611	0.82	0.08 to 8.88	0.869
III	2.73	0.34 to 21.79	0.344	0.96	0.1 to 9.06	0.97
IV	4.42	0.48 to 40.69	0.189	1.75	0.15 to 20.18	0.654
Aetiology of primary fistula						
Denovo	Ref					
Following abscess drainage	0.79	0.34 to 1.82	0.583			
Time from fistula diagnosis till surgery, months	0.98	0.87 to 1.12	0.815			
Sphincter defect	1.56	0.59 to 4.16	0.372			
Number of tracts						
Single and unbranched	Ref					
Single and branched	0.98	0.35 to 2.76	0.976			
Position of internal opening						
Below dentate line	Ref					
At dentate line	0.97	0.26 to 3.59	0.963			
Above dentate line	2.22	0.56 to 8.81	0.258			
Position of external opening						
Anterior	Ref					
Posterior	1.82	0.68 to 4.85	0.231			
Right lateral	1.09	0.27 to 4.35	0.901			
Left lateral	1.6	0.45 to 5.66	0.469			
Horseshoe extension	1.1	0.39 to 3.09	0.858			
Distance between external opening and anal verge, cm	0.78	0.48 to 1.27	0.316			
Type of trans-sphincteric fistula						
Low	Ref					
Mid	0.76	0.14 to 4.06	0.751			
High	1.11	0.31 to 4	0.87			
Length of tract, cm	0.76	0.46 to 1.26	0.283			
Fistula diameter, mm	0.39	0.18 to 0.84	**0.015**	0.6	0.26 to 1.41	0.242
Draining seton	2.52	0.97 to 6.53	0.057	1.03	0.28 to 3.7	0.968
Time from seton insertion till surgery, weeks	0.97	0.81 to 1.16	0.765			
Operative time, min	0.97	0.92 to 1.02	0.198	1.01	0.94 to 1.09	0.841
Clavien-Dindo classification	0.72	0.37 to 1.4	0.333			
Postoperative VAS	1.03	0.84 to 1.27	0.752			
High postoperative mean resting anal pressure, mmHg	0.82	0.74 to 0.9	** < 0.001**	0.97	0.85 to 1.1	0.619
High postoperative mean squeeze anal pressure, mmHg	0.96	0.95 to 0.98	** < 0.001**	0.97	0.95 to 0.99	**0.001**
Procedure						
MAF	Ref			Ref		
LIFT	2.134	1.71 to 2.65	** < 0.001**	2.089	1.006 to 4.33	**0.04**

Statistical significance at *P*-value < 0.05

*VAS* visual analogue scale, *OR* odds ratio, *CI* confidence interval

## Discussion

PAF forms a significant part of the workload of colorectal surgeons, which is a very distressing condition for elderly patients and negatively affects their quality of life [52]. Currently, evidence regarding the outcomes of LIFT and MAF in elderly populations is lacking. In the present study, we evaluated the incidence and risk factors of recurrence and incontinence in the surgical treatment of high TPAF in the elderly by comparing the LIFT and MAF methods. Despite being insignificant, a higher healing rate was observed in the MAF group (87.5% vs. 89.3% in the LIFT and MAF groups, respectively). We used strict criteria for complete fistula healing (MRI data) because the reliability of clinical examination alone is uncertain, and clinical closure may precede MRI healing [53], so MRI is a more reliable predictor of outcomes. Consequently, MRI was used to evaluate healing, and our results were reliable. Fortunately, an MRI was performed for all patients at the end of the study. The incontinence rate was statistically significantly better in the MAF group than in the LIFT group (13.2% and 5.8% in the LIFT and MAF groups, respectively). During this study, we were concerned that age may influence anal sphincters and anorectal function, perhaps leading to an increased prevalence of fecal incontinence, which could confound our findings [54]. Our results did not consider age to be a predictor of poor outcomes.

Advancements in minimally invasive surgical techniques have motivated researchers to develop novel treatment methods to reduce postoperative recurrence and fecal incontinence. Khan et al. used a plug/biological mesh with a 50% recurrence rate without postoperative fecal incontinence [8]. Wang et al. used platelet-rich plasma (PRP) with a cure rate of PRP alone (62.39%) and 83.12% when PRP was administered with other treatments [11]. Tang et al. [10] compared VAAFT with traditional surgical treatment and found that patients who underwent VAAFT experienced recurrence rates of 22.0% and 20.0% in the conventional group (*P* = 0.806), and the Wexner score in the VAAFT group was significantly lower than that in the conventional group (*P* < 0.001). Another study reported that the healing rate of VAAFT may reach 76% [17]. Torre et al. compared VAAFT and LIFT and found that the recurrence rates in the two groups were similar [18]. A previous FiLaC study showed that 30 of 68 (44.1%) fistulas healed. No cases of incontinence following FiLaC were observed [13]. Although the comparison between these emergent techniques and LIFT and MAF techniques was not our endpoint, our healing rate was higher than these emergent techniques, reflecting the superior role of surgery in treating high TPAF in the elderly. However, future well-designed studies can compare these emerging techniques with LIF or MAF in the treatment of elderly patients with high TPAF.

### Primary outcome: healing and recurrence

Preventing recurrence is essential in TPAF. The reported healing rate of MAFs ranges from 37 to 90% [20–26], whereas LIFT exhibits a healing rate of 37 to 95% [18, 28–33]. The follow-up duration, inclusion criteria, patient number, and type of PAF influenced these variations. The current study demonstrated high success rates for both approaches, although the differences were not statistically significant. This finding aligns with earlier research [55, 56]. The elevated healing rate in both techniques is ascribed to the high-volume centers for PAF surgery, a skilled group of surgeons, minimal inter-surgeon variability, and implementation of standardized operating protocols. This study identified additional technical factors that increased the healing rate in both approaches. Leakage of the tract after ligation (LIFT) or closure of the internal opening (MAF) was first confirmed. In MAF, the internal opening was occluded using multiple interrupted sutures to avoid retraction of the tract induced by increased pressure in the anal canal during defecation. Second, in the MAF technique, we effectively addressed an unusually high internal orifice location and evaluated the gap to be covered following mucosal excision using well-vascularized tissues and approximations without tension. Appropriate anal retractors were used to enhance the visibility of the internal opening or facilitate further dissection. Proximal mobilization is not constrained by insufficient visibility, bleeding control issues, excessive traction, or flap injuries. The base of the flap was designed to be wider than the tip to prevent ischemia in the distal region. Third, all patients received instructions to minimize fluid accumulation at the surgical site, adhere to postoperative bed rest, and refrain from bowel movements for 3 days. Fourth, we did not close the external wound of the LIFT to facilitate drainage and administration of antibiotics, either perioperatively or postoperatively. We assert that antibiotics mitigate postoperative edema and the accumulation of infected fluid [57] despite differing perspectives on the function of antibiotics [58]. The follow-up period was another factor that influenced the healing [59]. The recurrence rate increases over time, with late recurrences noted 7–8 months after surgery [60]. The 3-year follow-up period in our study facilitated a more thorough evaluation of recurrence rates.

Upon careful examination of the recurrences, 52.9% of LIFT recurrences were inter-sphincteric PAFs, whereas recurrent TPAF occurred in all patients after MAF. LIFT may be better than MAF since its “downstaging” allows fistulotomy. We believe that fistulotomy after LIFT often divides the internal sphincter with more incontinence. This makes LIFT downstaging less beneficial for elderly patients.

### Secondary outcome: incontinence

Yassin et al. documented a 6% incidence of mild incontinence following the LIFT method [35]. In one study, MAF repair showed no incontinence [20], whereas other investigations have reported higher rates [24, 26, 36, 61]. Our findings regarding incontinence status were markedly superior in LIFT than in MAF. The reduced occurrence of incontinence following the MAF technique is due to its preservation of blood supply to the sphincter, in contrast to the LIFT technique, which may compromise the blood supply or cause sphincter injury during ligation of the fistula tract, especially in cases of deep fistulas. Furthermore, in the MAF approach, we employed a long retractor to reveal a prominent internal opening, which was managed with slight anal dilation to preserve the sphincter function. Comparing the outcomes of the LIFT and MAF procedures for closure of high PAF in the elderly is generally difficult because patients may report functional impairment of the anal sphincter prior to surgery. We employed validated functional grading systems, preoperative and postoperative manometry, and endoanal ultrasonography for a comprehensive data assessment. Postoperative changes in the mean resting sphincteric pressure and mean squeeze pressure favored MAF.

The comparison between MAF and LIFT techniques in treating TPAF in the elderly requires a careful balance between functional outcomes and perioperative risks. While our findings suggest that MAF is associated with a lower incidence of incontinence compared to LIFT, it is essential to consider whether this advantage justifies the longer operative time and higher Clavien-Dindo Grade III complications. From a clinical perspective, the impact of postoperative complications classified as Clavien-Dindo Grade III must be evaluated regarding their management, reversibility, and long-term patient outcomes. Although MAF presents a higher rate of these complications, they are often manageable with appropriate interventions and do not necessarily translate into worse long-term functional impairment. Conversely, incontinence—even at a lower incidence—can significantly affect a patient’s quality of life, particularly given the social and psychological burden associated with fecal leakage. Furthermore, the longer operative time of MAF should be weighed against its potential benefits in reducing recurrence and preserving continence. In cases where sphincter preservation is a priority, particularly in patients with high-risk factors for incontinence, MAF may still be preferable despite the increased surgical complexity. Future studies with long-term follow-up could further elucidate whether these short-term risks translate into significant long-term disadvantages. Furthermore, Logistic regression confirmed that operative time and CD classifications were not risk factors associated with higher incontinence. Many patients prefer postoperative sphincter function over the higher cure rate provided by MAF compared with LIFT. Ellis et al. [62] revealed that most patients preferred sphincter preservation procedures despite a higher cure rate.

### Risk factors for recurrence and incontinence

Various predictors influence the healing rate of PAF [39]. The length of the fistula is a significant risk factor [63]. This finding is consistent with our results. The current study found no PAF cutoff length linked to a significant recurrence rate, as this was not the focus of our investigation. We believe that the longer the fistula, the more permanent the residual epithelialization and necrotic tissue and the higher the incidence of infection, treatment difficulty, and risk of recurrence. Curettage of the track was performed; however, the inflammatory granulation tissue was not eliminated from the longer fistula tracts, leading to recurrence. This aligns with the findings of Liu et al. [64]. Smoking status is recognized as a predictor of surgical failure [57], which is consistent with our findings; however, another study [65] indicated that smoking had no effect on surgical success. Smoking impairs oxygen transport and cellular metabolism through carbon monoxide and hydrogen cyanide, which hinders oxidative metabolism and reduces rectal mucosal perfusion in the mucosal flap, potentially resulting in diminished production of platelet-derived growth factors in surgical wounds. These explanations align with those documented in previous studies [66, 67]. We recommend smoking cessation prior to surgery for PAF, consistent with previous guidelines [68]. This study examined PAF in the elderly, revealing that age did not serve as a predictor of recurrence or incontinence, consistent with findings from a previous study [65]. Logistic regression analysis indicated that a low Charlson comorbidity index (≤ 5) and a low postoperative mean squeeze anal pressure were significantly associated with a reduced risk of incontinence, whereas the LIFT technique was associated with a significantly increased risk of incontinence.

## Strength and limitation

This study had several limitations. This was a retrospective study; the surgeon’s discretion in treatment and differences in the participants’ baseline characteristics possibly introduced selection bias. However, all surgeries were performed by experienced colorectal surgeons in standardized operating protocols and high-volume centers for PAF surgery. We performed logistic regression for most predisposing factors that may affect the outcome, making our results robust. Future studies are required to compare the elderly and young regarding high TPAF and between LIFT and MAF with emerging techniques (e.g., FiLaC, VAAFT) in managing TPAF in elderly patients. Future studies with larger sample sizes are needed to evaluate recurrence incidence in both approaches in elderly patients with TPAF.

Notwithstanding, the strengths of this study included the pre-and postoperative evaluation of both techniques with anal manometry, endoanal ultrasound, MRI, and functional score. Several risk factors for recurrence and incontinence were identified in this study. Additionally, long-term follow-up and objective evidence of sphincter dysfunction or healing (anal manometry and magnetic resonance imaging, respectively) were assessed in this study.

## Conclusion

The clinical implication of this study is that it adds a step to the management of high TPAF in the elderly. Our study indicates that MAF may be a superior alternative to LIFT in older patients with TPAF with a significantly lower incidence of incontinence than LIFT. The MAF technique demonstrated established efficacy, with 89.3% of the patients attaining complete healing. We advocate MAF as a clinically effective and less invasive treatment for TPAF in the elderly population.

## Supplementary Information

Below is the link to the electronic supplementary material.

Supplementary file1 (JPEG 189 KB)

## Data Availability

No datasets were generated or analysed during the current study.
