# Supplementary figures and images for: Mucosal advancement flap versus ligation of the inter-sphincteric fistula tract for management of trans-sphincteric perianal fistulas in the elderly: a retrospective study
Source: Int J Colorectal Dis. 2025 Mar 12;40(1):61. doi: 10.1007/s00384-025-04846-5 (PMC11903622; doi:10.1007/s00384-025-04846-5)

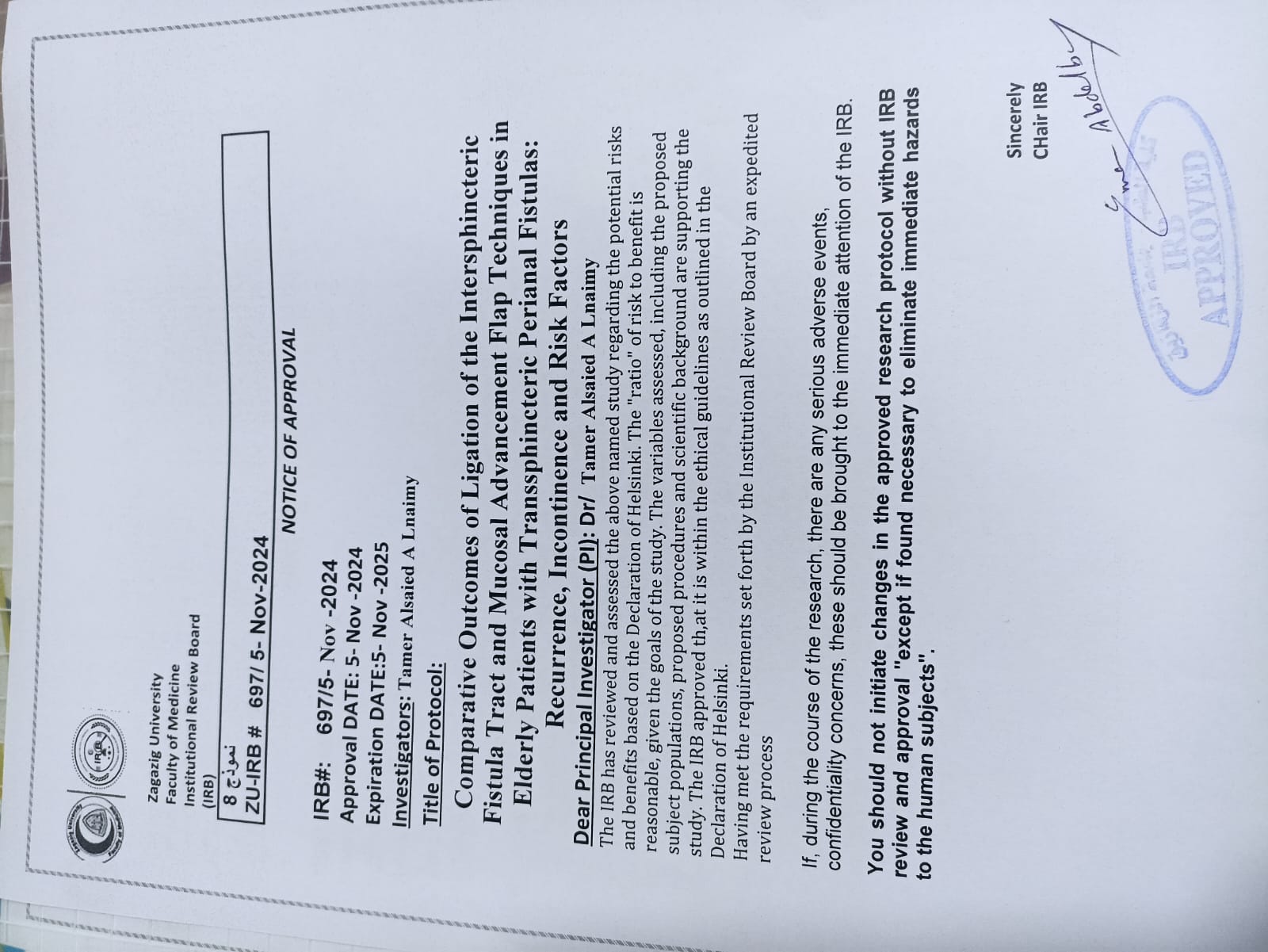

Supplement: Supplementary file 1 — Supplementary file1 (JPEG 189 KB) [file 384_2025_4846_MOESM1_ESM.jpeg]
